# Genome-wide characterization and expression profiling of the HD-ZIP gene family in Acoraceae under salinity and cold stress

**DOI:** 10.3389/fpls.2024.1372580

**Published:** 2024-04-26

**Authors:** Diyang Zhang, Xuewei Zhao, Ye Huang, Meng-Meng Zhang, Xin He, Weilun Yin, Siren Lan, Zhong-Jian Liu, Liang Ma

**Affiliations:** ^1^ Key Laboratory of National Forestry and Grassland Administration for Orchid Conservation and Utilization at College of Landscape Architecture and Art, Fujian Agriculture and Forestry University, Fuzhou, China; ^2^ College of Forestry, Fujian Agriculture and Forestry University, Fuzhou, China; ^3^ College of Biological Sciences and Technology, Beijing Forestry University, Beijing, China; ^4^ School of Pharmacy, Fujian Health Vocational and Technical College, Fuzhou, China

**Keywords:** Acoraceae, HD-ZIP gene family, salinity stress, low-temperature, expression pattern

## Abstract

The Homeodomain-Leucine Zipper (HD-ZIP) transcription factors play a pivotal role in governing various aspects of plant growth, development, and responses to abiotic stress. Despite the well-established importance of HD-ZIPs in many plants, their functions in Acoraceae, the basal lineage of monocots, remain largely unexplored. Using recently published whole-genome data, we identified 137 putative HD-ZIPs in two Acoraceae species, *Acorus gramineus* and *Acorus calamus*. These HD-ZIP genes were further classified into four subfamilies (I, II, III, IV) based on phylogenetic and conserved motif analyses, showcasing notable variations in exon-intron patterns among different subfamilies. Two microRNAs, *miR165/166*, were found to specifically target *HD-ZIP III* genes with highly conserved binding sites. Most *cis*-acting elements identified in the promoter regions of Acoraceae HD-ZIPs are involved in modulating light and phytohormone responsiveness. Furthermore, our study revealed an independent duplication event in *Ac. calamus* and a one-to-multiple correspondence between HD-ZIP genes of *Ac. calamus* and *Ac. gramineus.* Expression profiles obtained from qRT-PCR demonstrated that *HD-ZIP I* genes are strongly induced by salinity stress, while HD-ZIP II members have contrasting stress responses in two species. *HD-ZIP III* and *IV* genes show greater sensitivity in stress-bearing roots. Taken together, these findings contribute valuable insights into the roles of HD-ZIP genes in stress adaptation and plant resilience in basal monocots, illuminating their multifaceted roles in plant growth, development, and response to abiotic stress.

## 1 Introduction

The regulatory network that responds to abiotic and biotic stresses often involves transcription factors (TFs), which are key molecular regulators capable of activating or suppressing stress- responsive genes (Song et al., 2016). Therefore, gaining a nuanced understanding of TFs’ role in regulating stress responses is crucial for deciphering the stress signaling pathways and the underlying molecular mechanisms. The Homeobox (HB) gene family constitutes a vital group of TFs present in almost all eukaryotic organisms (Holland, 2013). These genes encode a conserved 60-amino-acid sequence known as the homeodomain (HD), which forms a helix-turn-helix structure, enabling it to bind specifically to target genes (Mukherjee et al., 2009). As a member of the homeobox gene family, Homeodomain-Leucine Zipper (HD-ZIP) is specific to plants and is characterized by the presence of a leucine zipper motif (LZ) located adjacent to the N-terminus of HD (Ariel et al., 2007). Based on domain similarity, gene structure and motif functions, HD-ZIP can be categorized into four subfamilies: I, II, III and IV (Mukherjee et al., 2009). HD-ZIP I and HD-ZIP II possess conserved HD and LZ domains. Additionally, HD-ZIP II features an extra conserved motif, CPSCE, positioned downstream of the LZ domain, along with a conserved N-terminal consensus sequence (Ariel et al., 2007). Both HD-ZIP III and HD-ZIP IV are featured by the presence of the START domain and the START adjacent domain. Notably, HD-ZIP III features a MEKHLA domain in the C-terminus (Schrick et al., 2004; Mukherjee and Burglin, 2006).

Members of HD-ZIP play an important role in regulating various aspects of plant growth, development, and responses to abiotic stimuli (Li et al., 2022). Most of the *HD-ZIP I* genes are actively involved in abscisic acid (ABA)-mediated signaling and abiotic stress tolerance (Gong et al., 2019). In *Arabidopsis*, the HD-ZIP I members *ATHB7* and *ATHB12* positively regulate *PP2C* and repress ABA receptors (*PYL5* and *PYL8*) in response to both drought stress and ABA (Valdés et al., 2012). Overexpression of the *AtHB13* gene confers cold stress tolerance by maintaining cellular stability (Gong et al., 2019). The sunflower genes *HaHB1* and *HB13* can enhance plant cold tolerance by promoting the production of proteins capable of stabilizing cell membranes and inhibiting ice growth (Cabello et al., 2012). In *Populus*, overexpression of *PsnHDZ63* can improve poplar’s salt tolerance by positively regulating peroxidase (POD) and superoxide dismutase (SOD) activities while reducing malondialdehyde (MDA) content (Guo et al., 2021). *HD-ZIP II* genes are mainly involved in shade avoidance regulation, adaxial polarity coordination, and auxin signaling (Sessa et al., 2018; He et al., 2020; Carabelli et al., 2021). *HAT1*, *HAT3*, *HAT3*, *ATHB2*, and *ATHB4* can all be induced when exposed to low red to far-red (R/FR) ratio light, suggesting redundancy in governing shade avoidance among HD-ZIP II members (Ciarbelli et al., 2008). Likewise, *HD-ZIP III* genes have been reported to control shade avoidance and auxin signaling (Müller et al., 2016; Merelo et al., 2017). They also subject to post-transcriptional regulation mediated by microRNAs *miR165/166*, leading to a negative impact on their expression through mRNA cleavage (Emery et al., 2003). HD-ZIP IV members have been shown to be specifically or preferentially expressed in plant epidermal or sub-epidermal cells, indicating their roles in transcriptional control of epidermal cell fate (Javelle et al., 2011). In addition, *HD-ZIP IV* genes are involved in biological processes such as anthocyanin accumulation, lipid transport, and cuticle biosynthesis (Chew et al., 2013).

In this study, we performed genome-wide identification, comparative analysis and expression profiling of HD-ZIP gene family in Acoraceae. Acoraceae represents a basal lineage of all other monocots (Givnish et al., 2018; Ma et al., 2023). It comprises a single genus *Acorus*, housing two accepted species, *Acorus calamus* (tetraploid) and *Ac. gramineus* (diploid), predominantly thriving in the humid areas of temperate, tropical, and subtropical regions (Ma et al., 2023). Given its unique phylogenetic position and strong stress resistance characteristics (Zhang et al., 2018; Zhou et al., 2018), the characterization of HD-ZIPs in Acoraceae, particularly in response to salinity and cold stress, could provide insights into the ancestral monocot gene toolkit for abiotic stress tolerance. Furthermore, this study holds the potential to reveal the interplay among HD-ZIP members and the regulatory network that underlies stress response.

## 2 Material and methods

### 2.1 Plant material treatment

A total of nine mature *Ac. gramineus* and *Ac. calamus* plants, all at an identical growth stage, were obtained from the botanical garden of Fujian Agriculture and Forestry University and subsequently transferred to an artificial climate culture chamber. During a week of cultivation, these plants experienced consistent conditions, including a photoperiod of 16 hours light/8 hours dark and a temperature range of 15°C to 25°C. Three plants, designated as control samples, were assigned as Group A. The remaining two groups, each consisting of three plants subjected to stress treatments, were designated as Group B and Group C. Group B plants were subjected to salinity stress, with irrigation involving a 200 mM NaCl solution at their roots (Zhang et al., 2020). Meanwhile, Group C plants experienced chilling stress, being placed under a temperature of 4°C (Shen et al., 2019). After a 72-hour post-treatment period, leaves and root samples (three replicates each) were collected from all experimental groups for subsequent analysis. In Group A, leaves and root samples were designated as Control-L and Control-R, respectively. In Group B, leaves and root samples were marked as NL and NR, and in Group C, as CL and CR, respectively.

### 2.2 Data sources

Genome sequences, annotation files, and raw data of transcriptome sequencing from different tissues of *Ac. calamus* and *Ac. gramineus* (accession number: PRJNA782402) were downloaded from National Center for Biotechnology Information (NCBI). HD-ZIP proteins of *Arabidopsis thaliana* were retrieved from TAIR (http://www.arabidopsis.org).

### 2.3 Identification and physicochemical properties of HD-ZIP genes

To identify HD-ZIP homologs in *Ac. calamus* and *Ac. gramineus*, a local BLASTp search was conducted using *A. thaliana* HD-ZIP proteins as the query. Two conserved HD-ZIP domains, namely homeodomain (PF00046) and the leucine zipper domain (PF02183) were downloaded from the online database (https://www.ebi.ac.uk/interpro/) (Finn et al., 2010) to perform an HMMER search with default parameters. Following the amalgamation of BLASTp and HMMER results, truncated and redundant proteins were manually eliminated. NCBI Batch CD Search Tool (https://www.ncbi.nlm.nih.gov/Structure/bwrpsb/bwrpsb.cgi) was employed to verify the presence of the HD-ZIP domain in candidate Acoraceae HD-ZIPs (AcHD-ZIPs). The completed protein sequences of AcHD-ZIPs were listed in **Supplementary Data Sheet**. The physicochemical properties and subcellular localization of HD-ZIP genes were predicted by ExPASy database (Artimo et al., 2012) and Plant-mPloc (Chou and Shen, 2010), respectively.

### 2.4 Phylogenetic analysis

The HD-ZIP protein sequences of *A. thaliana*, *Ac. calamus* and *Ac. gramineus* were aligned using MAFFT (Rozewicki et al., 2019). For the construction of the phylogenetic tree, the maximum likelihood (ML) method was employed through RAxML on the CIPRES Science Gateway web server (RAxML-HPC2 on XSEDE) (Miller et al., 2015). The analysis utilized the Protein CAT model, GTR matrix, and included 1000 bootstrap iterations to assess tree robustness. The resulting phylogenetic tree file was refined using Figtree (http://tree.bio.ed.ac.uk/software/figtree/) and Evolview (He et al., 2016).

### 2.5 Motif and gene structure analysis

Gene structure analysis was conducted using the GSDS tool (http://gsds.gao-lab.org/) (Hu et al., 2015). Identification of conserved motifs in HD-ZIP sequences was performed through the MEME online tool (http://meme-suite.org/tools/meme) (Bailey et al., 2009) with default parameters. TBtools (Chen et al., 2020) was employed for the visualization of both motifs and gene structure in HD-ZIP genes.

### 2.6 Gene location visualization, synteny analysis and selective pressure

The gene location visualization, synteny relationship, and the ratio of *K*a/*K*s (the number of non-synonymous substitutions per non-synonymous site (*K*a) to the number of synonymous substitutions per synonymous site (*K*s) of HD-ZIP genes) were analyzed using TBtools (Chen et al., 2020).

### 2.7 miRNA targeting prediction

To investigate potential target sites on AcHD-ZIPs for microRNA (miRNA) sequences, the web-based psRNA Target Server (https://www.zhaolab.org/psRNATarget/analysis, accessed on 20 December 2023) was employed with default parameters, where the expectation value was set to 5. A higher expectation value (penalty) indicates less similarity (and possibility) between miRNAs and their target candidates.

### 2.8 Protein-protein interaction

The STRING database (http://string-db.org) (Szklarczyk et al., 2023) was used to examine protein–protein interactions among AcHD-ZIPs. The homologous genes were paired based on the highest bit score, and the lines, distinguished by various colors, represent different types of evidence for protein interactions. Gene ontology (GO) functional annotation for the identified protein associations was performed using Blast2GO (Conesa et al., 2005).

### 2.9 Prediction of *cis*-acting elements

TBtools (Chen et al., 2020) was utilized to extract the 2000 base pairs upstream of all HD-ZIPs. PlantCARE (Lescot et al., 2002) (http://bioinformatics.psb.ugent.be/webtools/plantcare/html/) was employed for the identification and annotation of *cis*-acting elements present in the promoter regions. The numbers and responsive functions of *cis*-acting elements were visualized using TBtools (Chen et al., 2020).

### 2.10 Expression analysis

Transcript quantification and calculation of the FPKM (fragments per kilobase of transcript per million mapped reads) value for each gene were conducted using RSEM (Li and Dewey, 2011). Heatmaps based on the FPKM matrix were generated through TBtools (Chen et al., 2020).

To examine the role of AcHD-ZIPs in response to cold and salinity stress, we selected one member from each subfamily with the highest expression level for qRT-PCR analysis. Three replicates of leaves and roots from Groups A, B, C were sampled. Total RNA from these tissues was extracted using the FastQuant RT kit (Tiangen Biotech), with RNA concentration ranging from 81.9–442.5 ng/µl and A260/280 value ranging from 1.96–2.12, indicating high-quality extracted RNA. Specific PCR primers were designed and verified using the Primer3Plus online tool (http://www.primer3plus.com/cgi-bin/dev/primer3plus.cgi) and DNAMAN software (Lynnon Biosoft). Gene-specific primers for eight selected genes and their housekeeping genes were listed in **Supplementary Table 1**. The PrimeScriptTM RT reagent Kit (TaKaRa Bio) and TB GreenPremix Ex Taq II (TaKaRa Bio) were used for cDNA synthesis and qPCR, respectively. All experiments were performed in three biological replicates, each with three technical replicates. The relative gene expression was calculated using the 2^−ΔΔCT^ method.

## 3 Results

### 3.1 Identification of Acoraceae HD-ZIPs and their physicochemical properties

A total of 137 putative HD-ZIP genes, with 48 in *Ac. gramineus* and 89 in *Ac. calamus* were identified based on the presence of two conserved domains of HD-ZIP proteins. These HD-ZIP sequences demonstrated considerable variability in the number of amino acids (aa), spanning a range from 85 to 928. Correspondingly, their molecular weights (Mw) range from 11.99 to 131.3 kDa (**Supplementary Table 2**). Notably, the deduced grand average of hydrophilic (GRAVY) values for all HD-ZIP proteins was negative, indicative of pronounced hydrophilicity. Moreover, a majority of these proteins displayed an instability index (II) exceeding 40, signifying their inherent instability (Gasteiger et al., 2005). Results from subcellular location predictions consistently indicated that all HD-ZIP proteins were situated in the nucleus, suggesting a functional role within the nucleus akin to most transcription factors (**Supplementary Table 2**).

### 3.2 Phylogenetic tree of the HD-ZIP gene family

**Figure 1:**
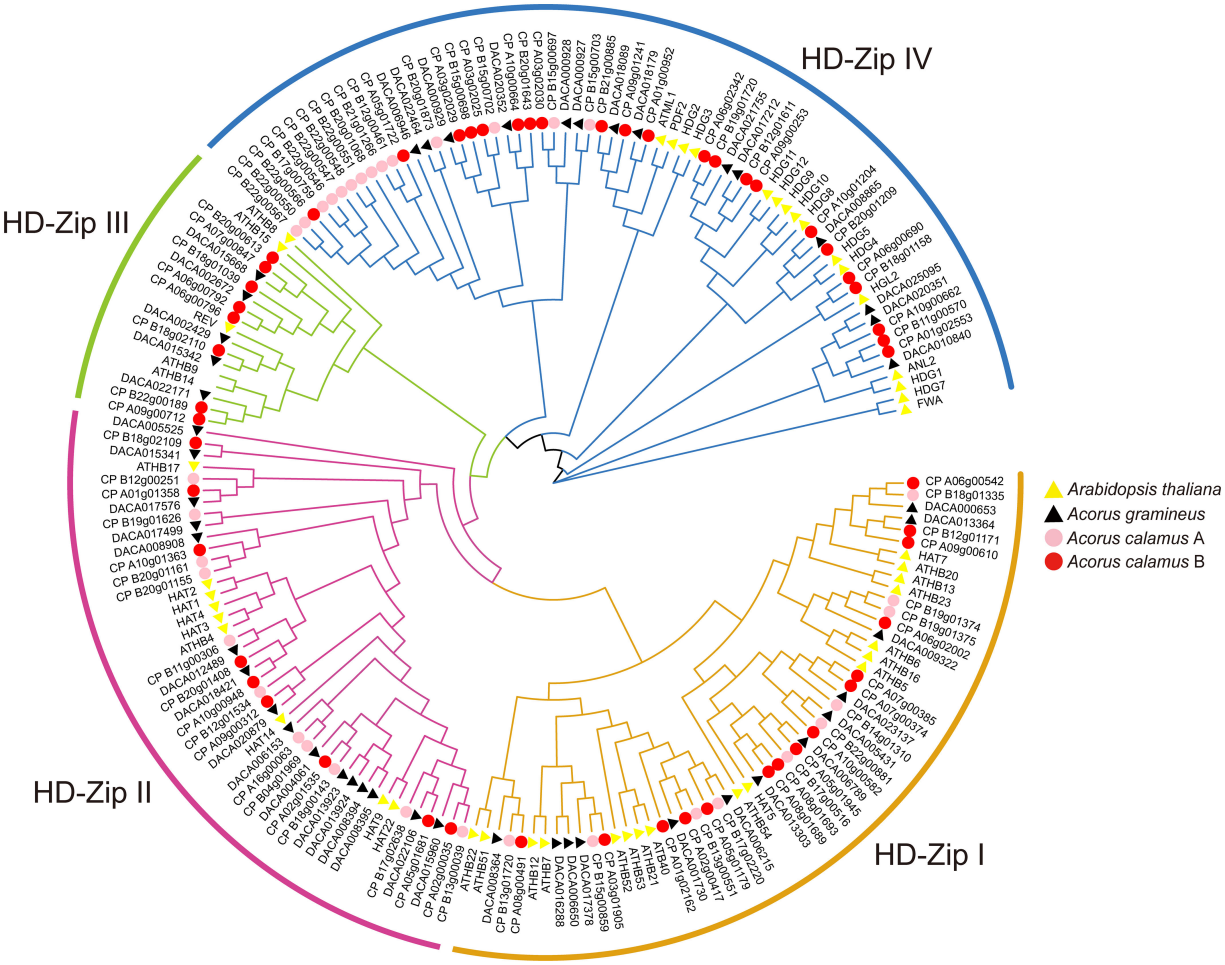
Phylogenetic tree of HD-ZIP based on the protein sequences of *Ac. gramineus*, *Ac. calamus* and *A. thaliana*. The HD-ZIP gene family was classified into four subfamilies: HD-ZIP I, HD-ZIP II, HD-ZIP III and HD-ZIP IV. HD-ZIP protein sequences of all species are available in **Supplementary Data Sheet**.

The phylogenetic tree has categorized the HD-ZIP genes into four subfamilies: HD-ZIP I, HD-ZIP II, HD-ZIP III, and HD-ZIP IV (**Figure 1**). This classification aligns with studies conducted in many other species (Wei et al., 2019; Yang et al., 2022). Within these subfamilies, HD-ZIP I comprises 13 and 25 members in *Ac. gramineus* and *Ac. calamus*, respectively. There are 16 and 20 HD-ZIP II members in *Ac. gramineus* and *Ac. calamus*, respectively. HD-ZIP III encompasses five and eight members in *Ac. gramineus* and *Ac. calamus*, respectively. HD-ZIP IV constitutes a sizable subfamily of genes, including 14 members in *Ac. gramineus* and 36 members in *Ac. calamus*.

### 3.3 Conserved motifs and gene structures of HD-ZIP proteins

**Figure 2:**
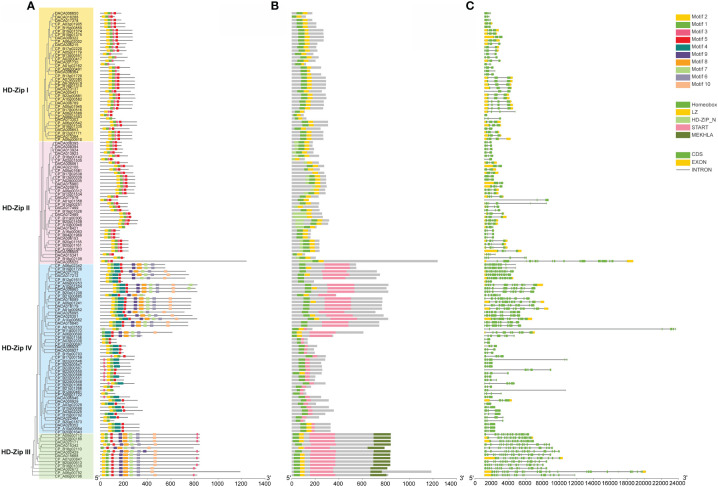
Conserved motifs, conserved domains and gene structure of AcHD-ZIPs. **(A)** Predicted motifs with phylogenetic tree. **(B)** Conserved domains. **(C)** Gene structure of HD-ZIPs, with green blocks, yellow blocks and grey lines representing coding regions (CDS), exons and introns, respectively.

Motifs of HD-ZIP proteins in Acoraceae were examined using the online analysis tool MEME, with an upper bound of 20 motifs. Among the identified motifs. Motif 2, motif 1, motif 3 and motif 5 demonstrated high conservation across all HD-ZIPs, whereas motif 4 was specifically distributed in HD-ZIP IV (**Figure 2A**). Motifs 9, 8, 7, 6, 10 were exclusive present in HD-ZIP III and HD-ZIP IV. In line with previous findings (Ariel et al., 2007), the MEKHLA domain is exclusively present in HD-ZIP III, and HD-ZIP II is characterized by a N-terminal consensus sequence (**Figure 2B**). To further explore the characteristics of HD-ZIPs in Acoraceae, an analysis of intron-exon structure was conducted. The results revealed that the *AcHD-ZIP* genes consist of 0–2 exons and 2–25 introns, displaying a noteworthy degree of variability in intron lengths and numbers. In general, most *HD-ZIP III* and *HD-ZIP IV* genes exhibited longer intron lengths compared to those in *HD-ZIP I* and *HD-ZIP II* (**Figure 2C**).

### 3.4 Chromosomal localization and gene duplication events of *AcHD-ZIP* genes

**Figure 3:**
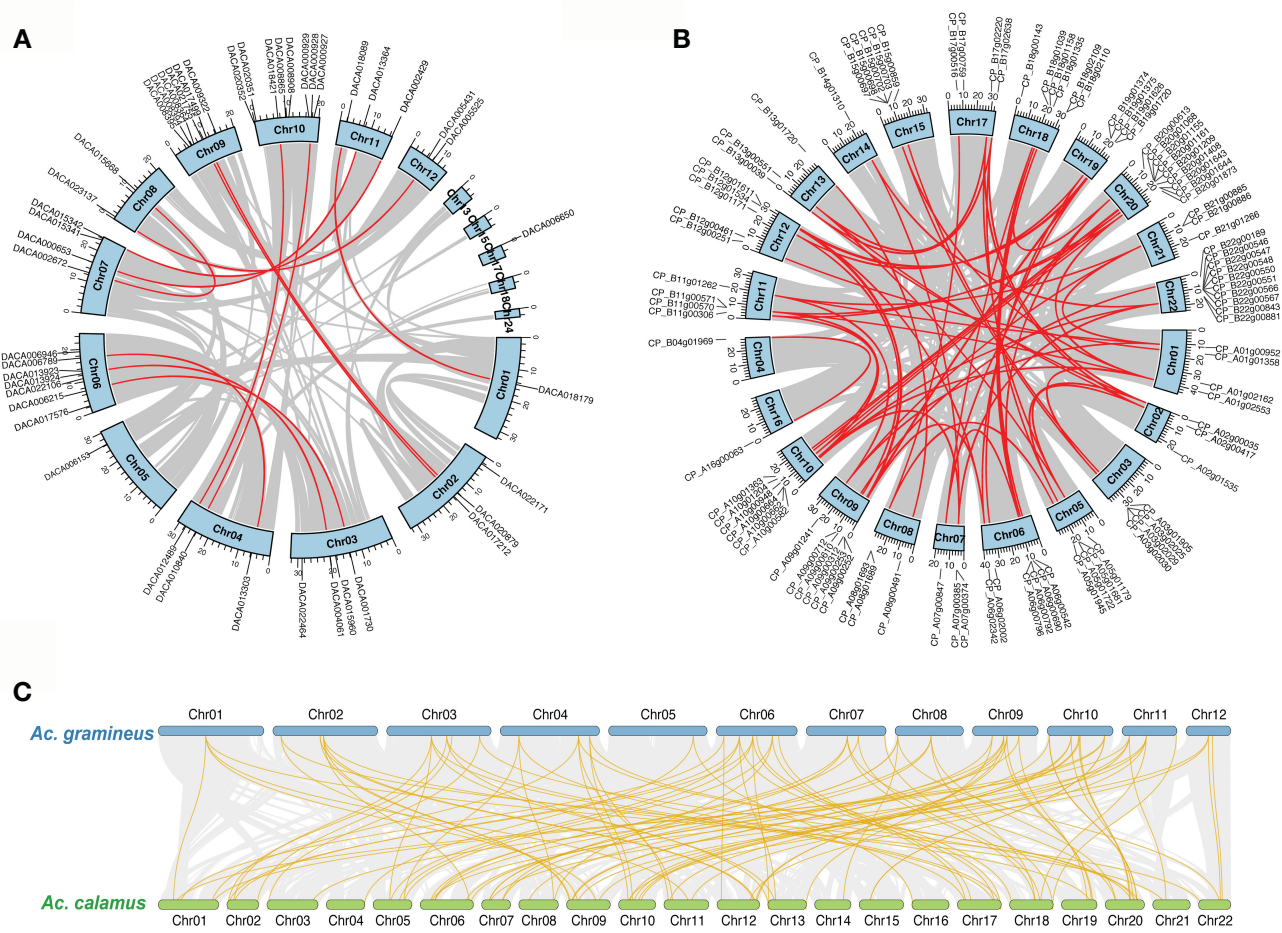
Chromosomal location and collinearity of HD-ZIP family genes. **(A)** Chromosomal location and self-collinearity in *Ac. gramineus.* **(B)** Chromosomal location and self-collinearity in *Ac. calamus.* Chromosomes are depicted by light blue boxes, with segmental duplication genes linked by red lines. **(C)** Intra-genomic collinearity between *Ac. gramineus* and *Ac. calamus* with HD-ZIP genes highlighted in gold.

We investigated the chromosomal localization for AcHD-ZIPs to identify potential duplication events. The result showed tandem duplications on Chr 10, Chr 6 and Chr 9 for *Ac. gramineus*, and on Chr 18, Chr 19 and Chr 22 for *Ac. calamus* (**Supplementary Figure 1**). In addition, synteny analysis revealed 12 and 63 pairs of segmental duplications for *Ac. gramineus* and *Ac. calamus*, respectively (**Figures 3A, B**). These gene pairs were selected for further selection pressure analysis. The results indicated that the *K*a/*K*s ratios of all HD-ZIP genes were less than one, with most values below 0.4, suggesting that all AcHD-ZIPs experienced strong purifying selection (**Supplementary Table 3**) (Zhang et al., 2006). The intra-genomic collinearity observed between the chromosomes of *Ac. gramineus* and *Ac. calamus* revealed a one-to-two or one-to-multiple correspondence of their HD-ZIP genes, suggesting that an independent duplication event has taken place in *Ac. calamus* (**Figure 3C**).

### 3.5 Targeting miRNAs and their binding sites for *AcHD-ZIP* genes

**Figure 4:**
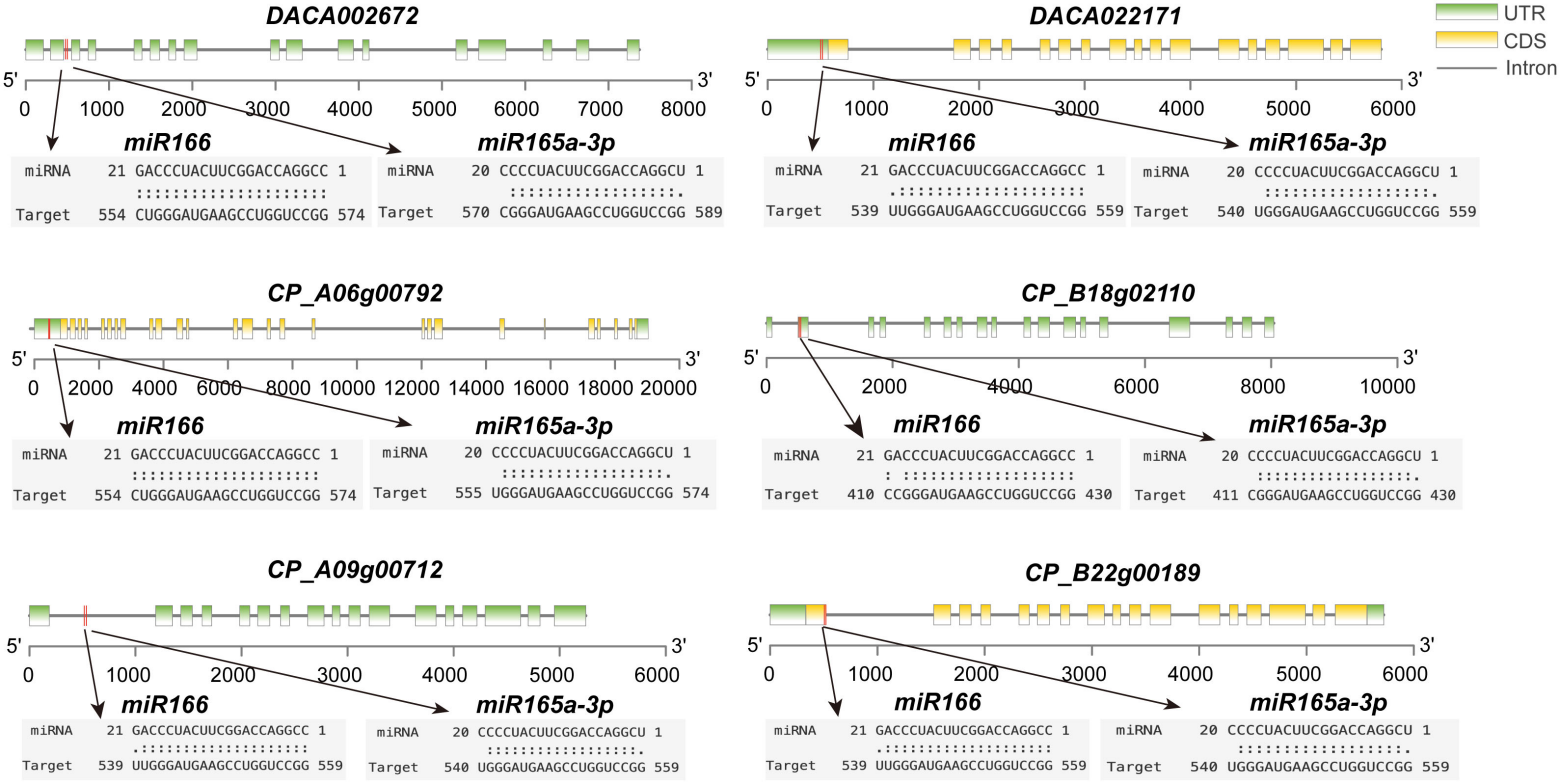
Some gene targets for *miR165* and *miR166* in *Ac. gramineus* and *Ac. calamus.* The coding region of HD-ZIP is highlighted in yellow boxes, and splicing sites are indicated by red lines.

The miRNA prediction results revealed that, within the narrowest expectation values (ranging from 0 to 1), *miR165* and *miR166* are the best-hit miRNAs (**Supplementary Table 4**). They also show a high degree of specificity in targeting *HD-ZIP III* genes. In addition, their binding sites in HD-ZIP III sequences are highly conserved. For instance, *miR166* predominantly targets a specific region between 539 bp and 574 bp, while *miR165* exhibits its primary targets within a range of 540 bp to 589 bp (**Figure 4**). This target specificity indicates the interaction between these miRNAs and *HD-ZIP III* genes is evolutionarily conserved.

### 3.6 Interaction network of AcHD-ZIP proteins

**Figure 5:**
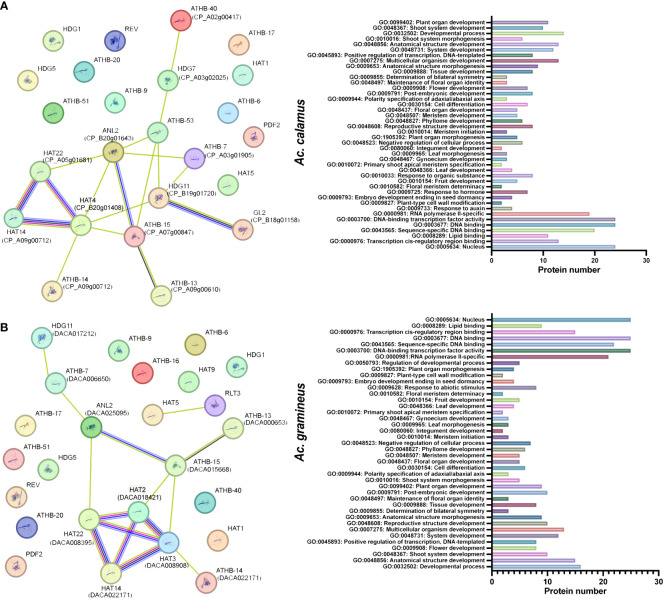
Protein interaction network and GO functions of AcHD-ZIP proteins based on the interactions of their orthologs in *Arabidopsis*. **(A)** Protein interaction network of *Ac. calamus.* **(B)** Protein interaction network of *Ac. gramineus.* The nodes represent different proteins, while the color-coded lines indicate various types of evidence for the interactions. Light blue lines, known interactions sourced from curated databases; Red lines, known interactions that have been experimentally determined. Blue lines, predicted interactions based on gene co-occurrence. Black lines, interactions derived from co-expression data. Green lines, interactions identified through text mining.

Interactions between HD-ZIP proteins of *Ac. gramineus* and *A. thaliana*, as well as between *Ac. calamus* and *A. thaliana*, were analyzed to predict the functional network of AcHD-ZIPs (**Figure 5**). In *Ac. calamus*, an interaction network involving 13 HD-ZIP proteins was identified. Notably, HAT14 (CP_A09g00712), HAT4 (CP_B20g01408) and HAT22 (CP_A05h01681) were found to be functionally linked within this network (**Figure 5A**). In *Ac. gramineus*, we detected an robust interconnected network comprising 10 HD-ZIP proteins, including HAT14 (DACA022171), HAT22 (DACA018421), HAT2 (DACA018421) and HAT3 (DACA008908). These associations were found to have diverse functions, including GO terms related to vegetative or reproductive organ development, response to hormone, and abiotic stimulus (**Figure 5B**). These findings suggest a potential synergy among these HD-ZIP II proteins, indicating their involvement in shared molecular pathways governing essential biological processes in plants.

### 3.7 *Cis*-acting regulatory elements in the promoter of *AcHD-ZIPs*

**Figure 6:**
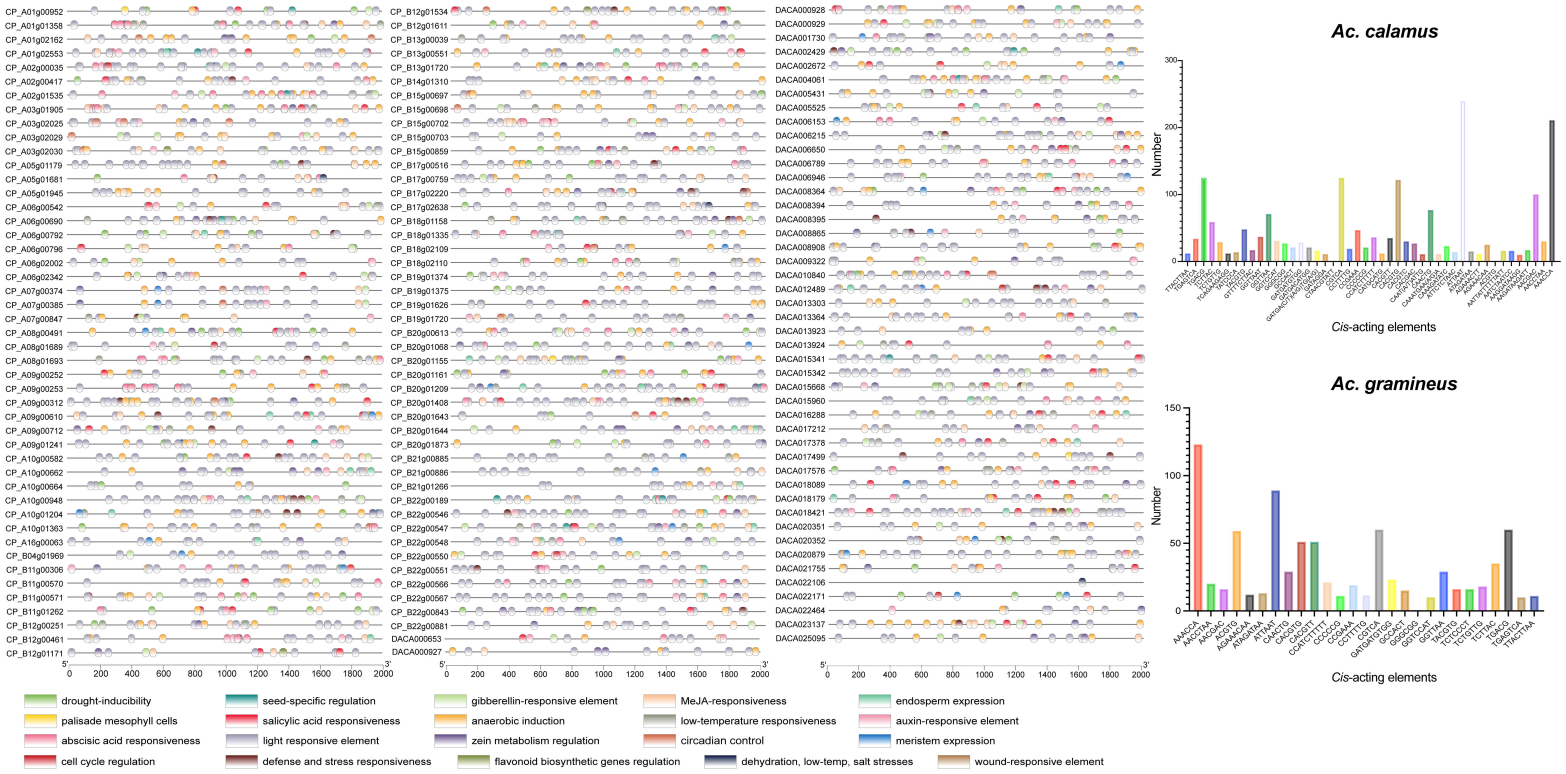
*Cis*-acting elements in the promoter regions of HD-ZIP genes. Elements with similar regulatory functions were displayed in the same color. Numbers of each type of elements were shown on the right side.

To investigate the regulatory functions of AcHD-ZIPs, we retrieved the 2,000 bp promoter regions of HD-ZIP genes to identify putative *cis*-acting elements. In total, 2,931 *cis*-acting elements, comprising 73 types and 20 responsive functions, were identified for both *Ac. gramineus* and *Ac. calamus* (**Figure 6**; **Supplementary Table 5**). The most abundant elements in *Ac. gramineus* and *Ac. calamus* were AAACCA (14.52%) and ATTAAT (11.52%), respectively (**Figure 6**; **Supplementary Table 6**). The identified *cis*-acting elements displayed various functions, including phytohormone responsiveness (gibberellin, auxin, methyl jasmonate (MeJA), salicylic acid, and ABA), stress responsiveness (drought, anoxic, wound, anaerobic, defense, dehydration, low-temperature, and salt). These elements are also associated with plant growth and development, such as light response, cell cycle regulation, and circadian control (**Figure 6**). Elements associated with light responsiveness are the most prevalent and distributed across all AcHD-ZIPs. Each *HD-ZIP* gene harbors multiple types of these elements, supporting the key roles of HD-ZIP in modulating the phytohormone network and facilitating plant growth adaptation to environmental stimuli (**Figure 6**).

### 3.8 Tissue expression profiling and expression patterns of *AcHD-ZIP* genes in response to salinity and cold stress

**Figure 7:**
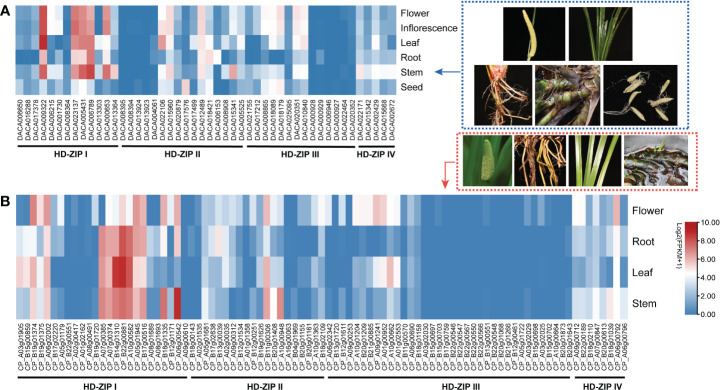
The expression profile of HD-ZIP genes among different tissues based on transcriptomic data. **(A)** *Ac. gramineus.* **(B)** *Ac. calamus.*.

**Figure 8:**
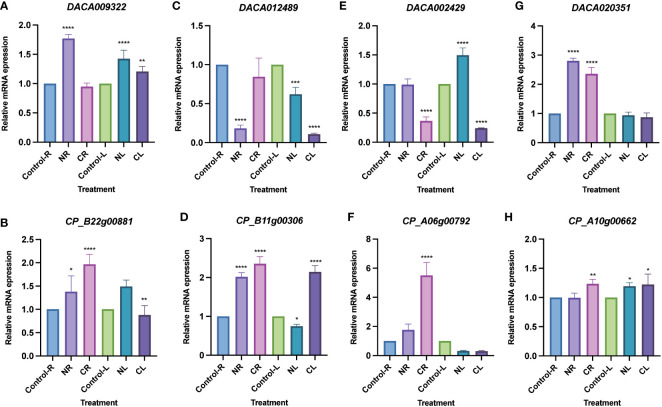
Expression profiles of *AcHD-ZIP* genes in leaves and roots under different abiotic stress treatments by qRT-PCR. **(A)** HD-ZIP I gene of *Ac. gramineus*. **(B)** HD-ZIP I gene of *Ac. calamus*. **(C)** HD-ZIP II gene of *Ac. gramineus*. **(D)** HD-ZIP II gene of *Ac. calamus*. **(E)** HD-ZIP III gene of *Ac. gramineus*. **(F)** HD-ZIP III gene of *Ac. calamus*. **(G)** HD-ZIP IV gene of *Ac. gramineus*. **(H)** HD-ZIP IV gene of *Ac. calamus*. Control-R and Control-L designate roots and leaves without stress treatment. NR and NL indicate roots and leaves subjected to treatment with a 200 mM NaCl solution. CR and CL represent roots and leaves exposed to a temperature treatment of 4°C. ANOVA multiple comparison test was performed with star marks *, **, *** and **** representing adjusted P<0.05, P<0.01, P<0.001 and P<0.0001, respectively (**Supplementary Table 7**).

Expression analysis was conducted using transcriptome data from *Ac. gramineus* and *Ac. calamus* tissues, including flowers, leaves, stems, roots, and seeds. The expression profile revealed a striking pattern in *AcHD-ZIP I* genes, which showed remarkably high expression levels in both reproductive and vegetative tissues, surpassing those of other subfamilies (**Figure 7**). For instance, members of the HD-ZIP I gene family, such as *DACA009322* and *CP_B22g00881*, exhibited consistently high expression across nearly all tissues. This robust and widespread expression implies an active and crucial involvement of *HD-ZIP I* genes in various plant organs, highlighting their significance in orchestrating developmental processes and responses to environmental cues. To further investigate the specific roles of HD-ZIP genes in response to cold and salinity stress, we selected one member with the highest expression level from each subfamily to conduct qRT-PCR analysis (see methods). In *Ac. gramineus* and *Ac. calamus*, the expression of *HD-ZIP I* genes were significantly elevated in both NR and NL upon exposure to 200 mM NaCl treatment (**Figures 8A, B**). However, their *HD-ZIP I* genes exhibited different responses under 4°C temperature. In *Ac. gramineus*, HD-ZIP I was slightly downregulated in CR and upregulated in CL, whereas in *Ac. calamus*, HD-ZIP I was upregulated almost two-fold in CR and downregulated in CL. On the other hand, under both cold and salinity stress conditions, the HD-ZIP II gene of *Ac. gramineus*, *DACA012489*, showed notable downregulation in both leaves and roots (**Figure 8C**). In contrast, the expression of *CP_B11g00306* was elevated in all tissues under stress conditions, except in leaves subjected to NaCl treatment (**Figure 8D**). The HD-ZIP III gene, *DACA002429* was downregulated in both CR and CL in response to cold stress (**Figure 8E**). Conversely, a substantial increase in the expression of *CP_A06g00792*, the HD-ZIP III gene of *Ac. calamus*, was observed in CR (**Figure 8F**). Notably, they displayed distinct expression patterns in NL (**Figures 8E, F**). In the case of *HD-ZIP IV* genes, *DACA020351* appears to play a role in the roots of *Ac. gramineus* under both salinity and cold stress, while the HD-ZIP IV gene of *Ac. calamus* maintained relatively stable expression levels under these stress conditions (**Figures 8G, H**).

## 4 Discussion

As a basal monocot lineage, Acoraceae species have evolved remarkable adaptations to their environment and possess strong stress resistance characteristics (Zhang et al., 2018; Zhou et al., 2018). Therefore, understanding the genetic basis and mechanisms underlying stress resistance in Acoraceae is crucial for unraveling the ancestral monocot gene toolkit for abiotic stress tolerance. The HD-ZIP gene family serves as a pivotal regulator in plant growth and development, significantly enhancing plant tolerance to various stresses (Sharif et al., 2021). However, current studies on the function and evolution of HD-ZIP genes are critically lacking in basal monocots.

A recent publication on the chromosome-scale assembly of the genomes of two Acoraceae species has provided opportunities to investigate the role of HD-ZIPs in stress responses.

### 4.1 The evolutionary dynamics of HD-ZIP gene family

Evolutionary novelty is thought to arise from gene duplication, a fundamental mechanism that enables duplicated genes to diverge and acquire new functions over time (Van de Peer et al., 2017). In this study, we identified a total of 137 HD-ZIP genes in Acoraceae, with 48 in *Ac. gramineus* and 89 in *Ac. calamus*. The one-to-multiple correspondence of HD-ZIP genes between the two Acoraceae species suggests a self-duplication event in *Ac. calamus* (**Figure 3C**). Indeed, tandem and segmental duplications are prevalent in *Ac. calamus* HD-ZIP genes (**Figure 3B**; **Supplementary Figure 1**). This may have been caused by gene duplications stemming from an independent whole-genome duplication (WGD) event (Ma et al., 2023). Interestingly, HD-ZIP genes in eudicots, such as cassava (Ding et al., 2017), *Arabidopsis* (Henriksson et al., 2005) and sesame (Wei et al., 2019), have evolved primarily through segmental duplication rather than tandem duplications. However, multiple tandem duplications of HD-ZIP genes, particularly HD-ZIP IV, were observed in both *Ac. calamus* and *Ac. gramineus* (**Supplementary Figure 1**). Evidence from phylogenetic studies suggests that gene duplications occurred recurrently in HD-ZIP IV homologs during the diversification of land plants (Zalewski et al., 2013). Therefore, these specific tandem duplications in *HD-ZIP IV* genes may facilitate the sub- or neo-functionalization of these genes, thereby driving the evolution of HD-ZIP gene family in basal monocots.

Variation in gene structure stands as a representative indicator of gene family evolution (Chen et al., 2022). In this case, we observed a significant degree of variability in intron lengths and numbers among different subfamilies (**Figure 2C**). These specific intron gains or losses may lead to the functional divergence among these subfamily members. Notably, longer introns were found in HD-ZIP III and IV compared to the other two subfamilies. This pattern is consistent with findings in many other species, such as sesame (Wei et al., 2019), *Prunus mume* (Li et al., 2019), and peach (Wang et al., 2023). Longer introns are favored in the course of gene evolution, as it promotes the efficiency of natural selection by enhancing recombination between two adjacent exons (Jo and Choi, 2015). In the context of HD-ZIP III and IV genes, longer introns may have profound implications for their early diverging roles in influencing plant growth and development (Prigge and Clark, 2006; Zalewski et al., 2013).

### 4.2 The promoter regions of *AcHD-ZIP* genes contain abundant stress-response elements

Transcription factors play an essential role in the abiotic stress response by regulating a large spectrum of downstream target genes via interaction with the *cis*-acting elements on the promoters of the genes (Hernandez-Garcia and Finer, 2014). These elements serve as the binding targets for upstream regulatory genes, ensuring the proper spatiotemporal pattern of gene expression (Marand et al., 2023). In this study, we identified a variety of regulatory elements in the promoter regions of AcHD-ZIPs (**Figure 6**). Previous research on drought- and salt-tolerance genes in *Arabidopsis* has revealed the presence of various *cis*-elements such as salicylic acid, ABA, GA, MeJA, and drought response elements in their promoter zones (Shariatipour and Heidari, 2018). Similarly, we have observed a significant proportion of *cis*-elements associated with phytohormone and stress responsiveness in *AcHD-ZIP* genes (**Supplementary Table 5**). For instance, many of these elements show responsiveness to ABA (ABRE), a crucial plant stress hormone. Given that HD-ZIP genes are actively involved in modulating ABA synthesis to enhance salt and drought tolerance (Wang et al., 2017; Gong et al., 2019; Jiao et al., 2024), it is plausible that these ABRE-contained genes could play a pivotal role in ABA-mediated signaling responding to salinity and drought stress in Acoraceae. In addition, we have identified elements directly associated with stress responses such as low temperature, salt and drought (**Supplementary Table 5**). The diverse stress-response functions of these *cis*-acting elements further underscore the pivotal roles of AcHD-ZIPs as key stress-responsive regulators.

### 4.3 HD-ZIP members play different roles in salinity and cold resistance

Cold and salinity, recognized as two adverse stress factors, have detrimental effects on plant growth and productivity (Mahajan and Tuteja, 2005). The HD-ZIP gene family is known to actively participate in responding to these two stresses (Li et al., 2022). Our tissue expression profile indicated broad expression of HD-ZIPs in both vegetative and reproductive tissues, with most *HD-ZIP I* genes displaying robust expression (**Figure 7**). Intriguingly, these HD-ZIP genes react differently in leaves and roots when exposed to NaCl treatment and a 4°C temperature condition (**Figure 8**). Previous studies have revealed that *HD-ZIP I* genes enhance salt stress tolerance by enhancing ion homeostasis, osmotic stress and antioxidant capacity, and through ABA-mediated signaling (Wang et al., 2017; Gong et al., 2019; Zhao et al., 2021). In *Ac. gramineus* and *Ac. calamus*, we observed the significant upregulation of *HD-ZIP I* genes in both roots and leaves when exposure to 200 mM NaCl treatment (**Figures 8A, B**), suggesting that HD-ZIP I genes were strongly induced by salinity stress. Although functional characterization is limited, several expression-based studies have suggested a role for *HD-ZIP II* genes in responding to salt and low-temperature stress (Yang et al., 2022). In *Arabidopsis*, HD-ZIP II genes including *HAT1*, *HAT2*, *HAT3*, *HAT4*, and *HAT22* can be strongly induced in response to NaCl stress, while *HAT9* was only induced by cold temperature (Perotti et al., 2021). In our study, the expression of the *HD-ZIP II* gene (*CP_B11g00306*) in *Ac. calamus* was elevated in almost all tissues under the two types of stress conditions (**Figure 8D**). However, the *HD-ZIP II* gene of *Ac. gramineus*, *DACA012489*, demonstrated significant downregulation in NR, CR, NL and CL (**Figure 8C**). Although these two genes form a sister clade in phylogenetic tree (**Figure 1**), these contrasting responses suggest functional divergence of HD-ZIP II members in *Ac. calamus* and *Ac. gramineus* under stress conditions. Despite *HD-ZIP III* and *HD-ZIP IV* genes have predominant role in plant growth and developmental processes (Ariel et al., 2007), a substantial increase in the expression of *HD-ZIP III* and *HD-ZIP IV* gene was observed in both NR and CR of *Ac. calamus* and *Ac. gramineus* (**Figures 8F, G**). This suggests that these two subfamily members are more sensitive to stress-bearing roots in Acoraceae. Several transgenic and silencing experiment of *HD-ZIP III* genes have validated their ability to enhancing plant salinity tolerance (Sharif et al., 2020; Hong et al., 2021). Characterization of loss/gain-of-function mutants and physiological experiments on overexpressed phenotypes can provide further insights into the roles of *HD-ZIP III* and *IV* genes in abiotic stress response.

## 5 Conclusions

Members of the HD-ZIP gene family serve as versatile regulators, influencing various aspects of plant growth and development. A total of 137 *AcHD-ZIP* genes were identified and categorized into four subfamilies by phylogenetic analysis, with HD-ZIP IV exhibiting a higher number of members. Motif and gene structure analyses unveiled significant variability among different subfamilies of AcHD-ZIPs, with HD-ZIP III and IV showing longer introns compared to HD-ZIP I and II. Collinear analysis unraveled a one-to-multiple correspondence between *Ac. gramineus* and *Ac. calamus*, with all HD-ZIP genes experiencing strong purifying selection. We also showed that the specific functions of *cis*-acting element in regulating and mediating phytohormone-induced stress. Furthermore, the expression patterns revealed by qRT-PCR data have shown distinct responses to salt and cold stress within and between different subfamily members. In particular, *HD-ZIP I* genes in both species show strong induction in response to salinity stress, which can be further validated in follow-up studies using functional and physiological approaches. Our study presents a comprehensive analysis to uncover the function and expression patterns of HD-ZIP genes in Acoraceae, laying the groundwork for a deeper understanding of how these HD-ZIP members contribute to in plant stress tolerance.

## Data availability statement

The original contributions presented in the study are included in the article/**Supplementary Material**. Further inquiries can be directed to the corresponding authors.

## Author contributions

DZ: Conceptualization, Data curation, Formal analysis, Writing – original draft, Writing – review & editing. XZ: Data curation, Methodology, Writing – review & editing. YH: Data curation, Writing – review & editing. MZ: Data curation, Writing – review & editing. XH: Methodology, Writing – review & editing. WY: Supervision, Writing – review & editing. SL: Supervision, Writing – review & editing. ZL: Conceptualization, Supervision, Writing – review & editing. LM: Funding acquisition, Resources, Writing – review & editing.
