# Supplementary material for: Genome-wide characterization and expression profiling of the HD-ZIP gene family in Acoraceae under salinity and cold stress
Source: Front Plant Sci. 2024 Apr 26;15:1372580. doi: 10.3389/fpls.2024.1372580 (PMC11082295; doi:10.3389/fpls.2024.1372580)
Supplement: Supplementary file 2 [file DataSheet_2.docx]

**Supplementary Information**

**Genome-wide characterization and expression profiling of the HD-ZIP gene family in Acoraceae under salinity and cold stress**

Diyang Zhang^1*^, Xuewei Zhao^1,2*^, Ye Huang^1^, Meng-Meng Zhang^1,2^, Xin He^1,2^, Weilun Yin^3^, Siren Lan^1^, Zhong-Jian Liu^1†^, Liang Ma^4†^

**Contents**

[Supplementary Figures 3](#_Toc156420544)

[Supplementary Figure 1. Chromosomal location of *HD-ZIP* genes. A. *Ac. gramineus.* B. *Ac. calamus.* 4](#_Toc156420545)

[Supplementary Tables 5](#_Toc156420546)

[Supplementary Table 1. The primers of eight *HD-ZIP* genes and their housekeeping genes. 5](#_Toc156420547)

[Supplementary Table 2. Physicochemical properties and subcellular location of *HD-ZIP* genes. 6](#_Toc156420548)

[Supplementary Table 3. *K*a/*K*s value of gene pairs. 10](#_Toc156420549)

[Supplementary Table 4. miRNA prediction for HD-ZIP genes (see separate file). 12](#_Toc156420550)

[Supplementary Table 5. *Cis*-acting element identified in the promotor region (see separate file). 12](#_Toc156420551)

[Supplementary Table 6. *Cis*-acting element types and numbers (see separate file). 12](#_Toc156420552)

[Supplementary Table 7. Statistical analysis using multiple comparisons for qPCR expression levels between two types of treatment. 13](#_Toc156420553)

# Supplementary Figures

**A**

**B**

## Supplementary Figure 1. Chromosomal location of *HD-ZIP* genes. A. *Ac. gramineus.* B. *Ac. calamus.*

# Supplementary Tables

## Supplementary Table 1. The primers of eight *HD-ZIP* genes and their housekeeping genes.

| **Genes** | **(5’–3’) Forward primer** | **(5’–3’) Reverse primer** |
| --- | --- | --- |
| *DACA011597* (housekeeping) | GACCACCTACAACTCCATCATGA | CAGTGATCTCCTTGCTCATACGA |
| *DACA009322* | GACGATCACCAGTACCAGTTTGA | AAATCCCACTCAATTCTCTCCGG |
| *DACA012489* | GCCGTTCGTGTTTCACCATAAG | CTCTTCCTCCAAATCCACCACC |
| *DACA002429* | CCATCCTCATGTCTTCAATCCCT | GTTTCTCCCATTTGTGGACTGTG |
| *DACA020351* | CGAGTTTTTCCCTGATGATGCTC | GATGCAAGATCCAATGTCCTGTG |
| *CP_B15g00839* (housekeeping) | GACCACCTACAACTCCATCATGA | CAGTGATCTCCTTGCTCATACGA |
| *CP_B22g00881* | CTCCATCAAGAACAACATCGACG | TATCTTCCCCAAATCCGTCCATC |
| *CP_B11g00306* | CCCCTTCAAACTAGCTCTCTGTT | CAGCTACTGAGGTTCCAGAATGA |
| *CP_A06g00792* | GGATGGATTGACTGGACTTGGAA | AATGCCCTCCTTCTCTGCTACTA |
| *CP_A10g00662* | TGGACAAACGCCTAGAATGTGA | TCTCGTAACCTGGGACTTGTTG |

## Supplementary Table 2. Physicochemical properties and subcellular location of AcHD-ZIPs.

| **Gene ID** | **AA^1^ (aa)** | **Mw^2^ (kDa)** | **pI^3^** | **II^4^** | **AI^5^** | **GRAVY^6^** | **Subcellular localization^7^** |
| --- | --- | --- | --- | --- | --- | --- | --- |
| DACA022171 | 841 | 91.48 | 6.13 | 44.46 | 85.39 | -0.139 | Nucleus. |
| DACA015342 | 842 | 91.9 | 5.64 | 46 | 88.28 | -0.071 | Nucleus. |
| DACA002429 | 840 | 91.78 | 5.86 | 46.43 | 87.31 | -0.1 | Nucleus. |
| DACA015668 | 838 | 92.24 | 5.84 | 49.83 | 88.57 | -0.108 | Nucleus. |
| DACA002672 | 813 | 89.26 | 5.87 | 45.99 | 89.85 | -0.091 | Nucleus. |
| DACA025095 | 712 | 78.16 | 5.96 | 52.39 | 81.6 | -0.336 | Nucleus. |
| DACA010840 | 743 | 81.7 | 5.84 | 50.38 | 83.32 | -0.276 | Nucleus. |
| DACA018179 | 766 | 83.89 | 5.78 | 44.24 | 82.34 | -0.293 | Nucleus. |
| DACA018089 | 770 | 84.49 | 5.7 | 843.56 | 84.19 | -0.269 | Nucleus. |
| DACA015341 | 114 | 12.89 | 4.21 | 51.57 | 97.54 | 0.181 | Nucleus. |
| DACA020352 | 330 | 36.46 | 5.22 | 61.65 | 68.85 | -0.672 | Nucleus. |
| DACA008865 | 806 | 88.65 | 5.76 | 47.34 | 77.56 | -0.339 | Nucleus. |
| DACA020351 | 781 | 84.94 | 5.86 | 52.57 | 80.38 | -0.316 | Nucleus. |
| DACA017212 | 750 | 81.96 | 6.42 | 47.02 | 79.09 | -0.3 | Nucleus. |
| DACA021755 | 724 | 79.89 | 6.12 | 47.85 | 78.92 | -0.327 | Nucleus. |
| DACA000928 | 216 | 24.62 | 5.01 | 48.97 | 76.76 | -0.63 | Nucleus. |
| DACA000929 | 314 | 35.15 | 7.71 | 39.77 | 67.39 | -0.94 | Nucleus. |
| DACA000927 | 189 | 22.04 | 6.46 | 51.67 | 68.57 | -0.928 | Nucleus. |
| DACA008395 | 180 | 21.44 | 9.62 | 55 | 69.33 | -1.152 | Nucleus. |
| DACA008394 | 177 | 20.95 | 9.46 | 56.5 | 70.51 | -1.077 | Nucleus. |
| DACA013923 | 184 | 21.64 | 9.3 | 57.99 | 70.54 | -0.993 | Nucleus. |
| DACA006650 | 175 | 20.4 | 7.71 | 57.55 | 64.69 | -1.021 | Nucleus. |
| DACA017499 | 228 | 25.99 | 5.53 | 55.73 | 79.17 | -0.596 | Nucleus. |
| DACA006946 | 250 | 28.48 | 5.8 | 43.76 | 79.96 | -0.628 | Nucleus. |
| DACA017378 | 175 | 20.44 | 6.47 | 59.32 | 64.69 | -1.037 | Nucleus. |
| DACA022106 | 292 | 30.64 | 8.7 | 58.31 | 60.21 | -0.769 | Nucleus. |
| DACA000653 | 245 | 27.91 | 6.27 | 64.92 | 64.98 | -0.931 | Nucleus. |
| DACA015960 | 292 | 32.51 | 8.16 | 58.31 | 60.21 | -0.769 | Nucleus. |
| DACA006789 | 272 | 30.65 | 4.68 | 69.82 | 64.52 | -0.855 | Nucleus. |
| DACA023137 | 290 | 33.15 | 4.71 | 67.16 | 70.66 | -0.836 | Nucleus. |
| DACA013364 | 267 | 30.44 | 6.84 | 71.79 | 74.61 | -0.701 | Nucleus. |
| DACA005431 | 256 | 29.6 | 4.69 | 59.38 | 66.29 | -0.992 | Nucleus. |
| DACA001730 | 203 | 23.87 | 6.77 | 60.54 | 69.66 | -0.992 | Nucleus. |
| DACA022464 | 232 | 26.49 | 9.72 | 58.81 | 71.47 | -0.906 | Nucleus. |
| DACA008364 | 159 | 18.28 | 9.37 | 61.77 | 66.29 | -0.784 | Nucleus. |
| DACA013924 | 190 | 22.42 | 9.04 | 53.58 | 66.16 | -1.042 | Nucleus. |
| DACA020879 | 297 | 32.61 | 5.94 | 63.17 | 59.9 | -0.705 | Nucleus. |
| DACA006153 | 160 | 18.67 | 9.74 | 78.81 | 71.31 | -1.115 | Nucleus. |
| DACA004061 | 231 | 26.04 | 6.9 | 49.91 | 65.84 | -0.818 | Nucleus. |
| DACA017576 | 229 | 25.6 | 8.09 | 71.98 | 61.75 | -0.91 | Nucleus. |
| DACA018421 | 211 | 22.96 | 9.09 | 68.53 | 65.31 | -0.861 | Nucleus. |
| DACA008908 | 237 | 26.86 | 9 | 57.47 | 67.13 | -0.951 | Nucleus. |
| DACA012489 | 258 | 27.81 | 4.86 | 58.52 | 70.27 | -0.725 | Nucleus. |
| DACA009322 | 273 | 30.61 | 4.71 | 64.23 | 58.61 | -0.769 | Nucleus. |
| DACA006215 | 218 | 25.69 | 7.05 | 58.14 | 64.82 | -1.071 | Nucleus. |
| DACA016288 | 121 | 14.29 | 6.37 | 51.24 | 74.21 | -0.831 | Nucleus. |
| DACA013303 | 126 | 14.58 | 4.88 | 71.28 | 73.41 | -0.723 | Nucleus. |
| DACA005525 | 1240 | 13.97 | 8.63 | 52.29 | 78.77 | -0.563 | Nucleus. |
| CP_A09g00712 | 842 | 91.41 | 6.06 | 44.19 | 85.17 | -0.138 | Nucleus. |
| CP_A07g00847 | 838 | 92.27 | 5.84 | 49.74 | 87.64 | -0.131 | Nucleus. |
| CP_A06g00792 | 1186 | 131.3 | 6.72 | 46.12 | 91.5 | -0.124 | Nucleus. |
| CP_A06g00796 | 820 | 89.89 | 5.96 | 45.73 | 90.05 | -0.074 | Nucleus. |
| CP_A01g02553 | 743 | 81.6 | 5.73 | 51.18 | 84.37 | -0.251 | Nucleus. |
| CP_A01g00952 | 766 | 83.88 | 5.81 | 43.8 | 82.72 | -0.289 | Nucleus. |
| CP_A09g01241 | 770 | 84.24 | 5.73 | 42.46 | 82.55 | -0.28 | Nucleus. |
| CP_A09g00253 | 188 | 22.13 | 6.66 | 56.61 | 52.45 | -1.035 | Nucleus. |
| CP_A10g01204 | 821 | 90.38 | 5.78 | 47.07 | 79.09 | -0.338 | Nucleus. |
| CP_A10g00664 | 329 | 36.35 | 5.3 | 59.42 | 67.26 | -0.671 | Nucleus. |
| CP_A10g00662 | 819 | 88.74 | 5.92 | 52.29 | 79.62 | -0.292 | Nucleus. |
| CP_A06g00690 | 608 | 67.42 | 6.43 | 50.41 | 81.78 | -0.296 | Nucleus. |
| CP_A06g02342 | 546 | 60.84 | 5.88 | 49.88 | 73.97 | -0.449 | Nucleus. |
| CP_A03g02025 | 357 | 40.12 | 6.01 | 48.03 | 64.2 | -0.957 | Nucleus. |
| CP_A09g00610 | 268 | 30.5 | 6.14 | 75.73 | 73.62 | -0.703 | Nucleus. |
| CP_A10g00948 | 309 | 34.39 | 8.6 | 69.2 | 60.65 | -0.896 | Nucleus. |
| CP_A06g00542 | 307 | 34.84 | 6.18 | 68.88 | 62.35 | -0.832 | Nucleus. |
| CP_A08g00491 | 249 | 28.06 | 5.5 | 57.21 | 66.59 | -0.683 | Nucleus. |
| CP_A02g00035 | 292 | 32.57 | 7.67 | 56.6 | 63.87 | -0.71 | Nucleus. |
| CP_A03g01905 | 208 | 24.14 | 5.23 | 56.55 | 63.8 | -0.929 | Nucleus. |
| CP_A02g00417 | 228 | 26.8 | 8.98 | 61.59 | 66.71 | -1.043 | Nucleus. |
| CP_A09g00312 | 284 | 31.35 | 6.78 | 64.72 | 58.87 | -0.749 | Nucleus. |
| CP_A05g01945 | 272 | 30.62 | 4.71 | 61.16 | 64.15 | -0.84 | Nucleus. |
| CP_A07g00385 | 289 | 33.06 | 4.6 | 68.67 | 70.55 | -0.816 | Nucleus. |
| CP_A07g00374 | 290 | 33.21 | 4.64 | 70.45 | 70.31 | -0.829 | Nucleus. |
| CP_A10g00582 | 294 | 33.05 | 5.28 | 64.68 | 65 | -0.766 | Nucleus. |
| CP_A05g01179 | 218 | 25.64 | 7.05 | 57.26 | 64.4 | -1.071 | Nucleus. |
| CP_B04g01969 | 160 | 18.58 | 9.74 | 78.26 | 70.12 | -1.065 | Nucleus. |
| CP_A10g01363 | 235 | 26.5 | 9.06 | 56.53 | 59.79 | -1.002 | Nucleus. |
| CP_A05g01681 | 237 | 25.85 | 8.17 | 66.8 | 63.42 | -0.708 | Nucleus. |
| CP_A01g01358 | 145 | 16.51 | 9.57 | 66.86 | 65.86 | -0.91 | Nucleus. |
| CP_A02g01535 | 110 | 12.21 | 9.36 | 57.19 | 52.27 | -0.727 | Nucleus. |
| CP_A06g02002 | 270 | 30.16 | 4.81 | 66.6 | 57.81 | -0.744 | Nucleus. |
| CP_A08g01693 | 101 | 11.99 | 9.17 | 52.16 | 84.85 | -0.574 | Nucleus. |
| CP_A05g01722 | 123 | 14.85 | 10.25 | 67.84 | 69.76 | -1.078 | Nucleus. |
| CP_A03g02030 | 135 | 15.69 | 5.35 | 59.07 | 80.89 | -0.514 | Nucleus. |
| CP_A03g02029 | 205 | 22.59 | 8.33 | 53.37 | 69.51 | -0.647 | Nucleus. |
| CP_A01g02162 | 121 | 13.81 | 10.09 | 32.75 | 75.79 | -0.616 | Nucleus. |
| CP_A08g01689 | 110 | 12.94 | 5.45 | 88.77 | 54.09 | -1.039 | Nucleus. |
| CP_B22g00189 | 842 | 91.48 | 6.09 | 44.98 | 84.48 | -0.153 | Nucleus. |
| CP_B18g01039 | 838 | 92.01 | 5.87 | 45.56 | 89.5 | -0.096 | Nucleus. |
| CP_A07g00847 | 789 | 86.32 | 5.57 | 46.57 | 90.13 | -0.036 | Nucleus. |
| CP_B18g02110 | 838 | 92.18 | 5.77 | 47.5 | 89.26 | -0.098 | Nucleus. |
| CP_B20g01643 | 329 | 36.32 | 5.23 | 59.23 | 67.26 | -0.675 | Nucleus. |
| CP_B18g02109 | 203 | 23.5 | 4.48 | 53.92 | 91.28 | -0.376 | Nucleus. |
| CP_B20g01209 | 820 | 90.33 | 5.76 | 47.06 | 79.55 | -0.334 | Nucleus. |
| CP_B21g00885 | 377 | 42.6 | 5.82 | 47.13 | 69.07 | -0.615 | Nucleus. |
| CP_B11g00570 | 175 | 20.73 | 7.88 | 63.62 | 62.29 | -1.219 | Nucleus. |
| CP_B19g01720 | 548 | 61.02 | 5.72 | 48.39 | 73.87 | -0.46 | Nucleus. |
| CP_B12g01611 | 719 | 78.86 | 6.31 | 47.73 | 79.39 | -0.321 | Nucleus. |
| CP_B15g00698 | 315 | 35.24 | 8.19 | 39.56 | 65.97 | -0.989 | Nucleus. |
| CP_B18g01158 | 352 | 40.07 | 8.31 | 57.24 | 75.31 | -0.596 | Nucleus. |
| CP_B12g01171 | 268 | 30.41 | 6.32 | 70.64 | 78.69 | -0.645 | Nucleus. |
| CP_B22g00566 | 254 | 28.06 | 6.59 | 40.69 | 74.09 | -0.652 | Nucleus. |
| CP_B20g01408 | 316 | 34.98 | 8.83 | 66.44 | 63.32 | -0.827 | Nucleus. |
| CP_B22g00551 | 200 | 23.03 | 8.95 | 44.12 | 74.1 | -0.847 | Nucleus. |
| CP_B22g00547 | 250 | 28.25 | 6.75 | 43.84 | 78 | -0.641 | Nucleus. |
| CP_B22g00548 | 196 | 22.45 | 9.2 | 51.16 | 67.14 | -1.017 | Nucleus. |
| CP_B15g00703 | 195 | 22.47 | 6.67 | 49.13 | 67.95 | -0.895 | Nucleus. |
| CP_B22g00550 | 259 | 28.69 | 7.8 | 39.67 | 75.64 | -0.611 | Nucleus. |
| CP_B17g00759 | 289 | 32.99 | 8.04 | 52.81 | 74.22 | -0.655 | Nucleus. |
| CP_B22g00567 | 256 | 28.91 | 7.17 | 44.69 | 80.35 | -0.634 | Nucleus. |
| CP_B19g01626 | 236 | 26.73 | 5.96 | 61.45 | 83.94 | -0.601 | Nucleus. |
| CP_B13g01720 | 250 | 28.13 | 5.38 | 56.26 | 74.76 | -0.698 | Nucleus. |
| CP_B20g01068 | 286 | 32.65 | 9.07 | 46.63 | 70.59 | -0.761 | Nucleus. |
| CP_B17g02638 | 276 | 30.52 | 8.88 | 59.88 | 63.99 | -0.791 | Nucleus. |
| CP_B22g00546 | 284 | 32.25 | 8.68 | 44.61 | 82.01 | -0.571 | Nucleus. |
| CP_B13g00039 | 291 | 32.42 | 8.45 | 59.23 | 62.1 | -0.76 | Nucleus. |
| CP_B18g01335 | 308 | 35.02 | 6.25 | 67.99 | 61.2 | -0.885 | Nucleus. |
| CP_B12g01534 | 287 | 32.67 | 7.64 | 64.37 | 59.62 | -0.755 | Nucleus. |
| CP_B12g00461 | 114 | 13.15 | 6.41 | 44.15 | 63.25 | -0.979 | Nucleus. |
| CP_B14g01310 | 290 | 33.16 | 4.6 | 71.04 | 69.97 | -0.811 | Nucleus. |
| CP_B15g00859 | 208 | 24.2 | 5.11 | 60.05 | 61.87 | -0.886 | Nucleus. |
| CP_B17g02220 | 208 | 24.43 | 8.56 | 51.62 | 70.29 | -0.965 | Nucleus. |
| CP_B22g00881 | 288 | 32.67 | 4.64 | 61.72 | 65.69 | -0.887 | Nucleus. |
| CP_A16g00063 | 163 | 18.89 | 9.28 | 70 | 68.22 | -1.108 | Nucleus. |
| CP_B18g00143 | 231 | 26.11 | 6.47 | 50.64 | 67.1 | -0.838 | Nucleus. |
| CP_B12g00251 | 232 | 26.08 | 8.67 | 63.78 | 71.47 | -0.833 | Nucleus. |
| CP_B20g01155 | 235 | 26.4 | 8.74 | 55.93 | 64.38 | -1.008 | Nucleus. |
| CP_B20g01161 | 235 | 26.4 | 8.74 | 55.93 | 64.38 | -1.008 | Nucleus. |
| CP_B11g00306 | 263 | 28.09 | 4.8 | 60.74 | 67.49 | -0.656 | Nucleus. |
| CP_B17g00516 | 266 | 29.89 | 4.74 | 61.03 | 65.23 | -0.848 | Nucleus. |
| CP_B19g01374 | 269 | 30.04 | 4.7 | 64.12 | 59.48 | -0.762 | Nucleus. |
| CP_B19g01375 | 269 | 30.04 | 4.7 | 64.12 | 59.48 | -0.762 | Nucleus. |
| CP_B13g00551 | 183 | 21.64 | 9.1 | 59.61 | 71.91 | -1.045 | Nucleus. |
| CP_B20g01873 | 115 | 13.7 | 9.79 | 65.72 | 72.87 | -0.95 | Nucleus. |
| CP_B15g00702 | 283 | 31.48 | 5.69 | 42.92 | 63.39 | -0.863 | Nucleus. |
| CP_B15g00697 | 135 | 15.63 | 5.19 | 72.79 | 83.04 | -0.494 | Nucleus. |
| CP_B21g01266 | 163 | 18.87 | 6.1 | 47.84 | 78.96 | -0.672 | Nucleus. |

Note: ^1^: amino acid number; ^2^: molecular weight; ^3^: theoretical isoelectric point; ^4^: instability index; ^5^: aliphatic index; ^6^: grand average of hydrophobicity; ^7^: subcellular localization predicted by Plant-mPloc (Chou and Shen, 2010).

## Supplementary Table 3. *K*a/*K*s value of gene pairs.

| **Seq_1** | **Seq_2** | ***K*a** | ***K*s** | ***K*a/*K*s** |
| --- | --- | --- | --- | --- |
| *DACA015341* | *DACA002429* | 0.41083003 | 1.36513049 | 0.30094562 |
| *DACA002672* | *DACA015668* | 0.04081702 | 0.41299042 | 0.09883285 |
| *DACA010840* | *DACA000928* | 0.60282998 | NaN | NaN |
| *DACA018179* | *DACA018089* | 0.03635832 | 0.76233251 | 0.04769352 |
| *DACA017212* | *DACA021755* | 0.10397589 | 0.56681234 | 0.18343971 |
| *DACA020879* | *DACA017499* | 0.41326161 | 1.22329528 | 0.33782654 |
| *DACA000653* | *DACA013364* | 0.11650035 | 1.04487221 | 0.11149722 |
| *DACA013303* | *DACA006789* | 0.23354874 | 0.87419397 | 0.26715895 |
| *DACA023137* | *DACA005431* | 0.15927749 | 0.61709729 | 0.25810758 |
| *DACA001730* | *DACA006215* | 0.08164478 | 0.5619659 | 0.14528422 |
| *DACA004061* | *DACA013924* | 0.187053 | 0.80132375 | 0.23342999 |
| *DACA012489* | *DACA018421* | 0.20726288 | 0.67206602 | 0.30839661 |
| *CP_A09g00712* | *CP_B22g00189* | 0.00579433 | 0.0889043 | 0.06517493 |
| *CP_A06g00792* | *CP_B18g01039* | 0.01633331 | 0.05010365 | 0.32599037 |
| *CP_A07g00847* | *CP_B18g01039* | 0.03849397 | 0.41588864 | 0.09255836 |
| *CP_B18g01039* | *CP_B20g00613* | 0.04125672 | 0.4094502 | 0.10076126 |
| *CP_A06g00792* | *CP_A07g00847* | 0.05255282 | 0.44273172 | 0.11870128 |
| *CP_A07g00847* | *CP_B20g00613* | 0.00575417 | 0.02738467 | 0.21012372 |
| *CP_A06g00792* | *CP_B20g00613* | 0.05286161 | 0.44725126 | 0.1181922 |
| *CP_A01g02553* | *CP_A03g02030* | 0.53228474 | 2.46068341 | 0.21631582 |
| *CP_A01g02553* | *CP_A10g00662* | 0.09960351 | 0.80005094 | 0.12449646 |
| *CP_A01g02553* | *CP_B11g00570* | 0.1393521 | 0.36573592 | 0.38101837 |
| *CP_A01g02553* | *CP_B15g00697* | 0.54981398 | 2.25053986 | 0.24430315 |
| *CP_A01g02553* | *CP_B20g01643* | 0.67329362 | 1.92693826 | 0.3494111 |
| *CP_A01g00952* | *CP_A09g01241* | 0.03068361 | 0.75307685 | 0.04074432 |
| *CP_A01g00952* | *CP_B11g01262* | 0.00175029 | 0.14855885 | 0.01178181 |
| *CP_A01g00952* | *CP_B21g00885* | 0.04466935 | 0.7285268 | 0.06131463 |
| *CP_A09g01241* | *CP_B11g01262* | 0.02712813 | 0.81891209 | 0.03312704 |
| *CP_B11g01262* | *CP_B21g00886* | 0.15782972 | 3.50742014 | 0.04499881 |
| *CP_A09g01241* | *CP_B21g00885* | 0.0245015 | 0.17689773 | 0.13850657 |
| *CP_A10g00664* | *CP_B20g01643* | 0.01578313 | 0.03271547 | 0.48243646 |
| *CP_A10g01204* | *CP_B20g01209* | 0.0096258 | 0.08010087 | 0.12017099 |
| *CP_A06g02342* | *CP_A09g00253* | 0.08199069 | 0.43764559 | 0.18734494 |
| *CP_A09g00253* | *CP_B19g01720* | 0.07934984 | 0.40555514 | 0.19565734 |
| *CP_A10g00662* | *CP_B11g00570* | 0.3176968 | 1.93081547 | 0.16454022 |
| *CP_A10g00662* | *CP_B20g01644* | 0.00914002 | 0.08041359 | 0.11366267 |
| *CP_A06g00690* | *CP_B18g01158* | 0.03794715 | 0.16031244 | 0.23670749 |
| *CP_A06g02342* | *CP_B12g01611* | 0.11317676 | 0.51790599 | 0.21852761 |
| *CP_A06g02342* | *CP_B19g01720* | 0.0151722 | 0.08507695 | 0.17833499 |
| *CP_B12g01611* | *CP_B19g01720* | 0.10971355 | 0.48437194 | 0.22650682 |
| *CP_A09g00252* | *CP_B12g01611* | 0.0233427 | 0.11204438 | 0.20833444 |
| *CP_A03g02025* | *CP_B15g00698* | 0.13041187 | 0.36513528 | 0.35716043 |
| *CP_B11g00571* | *CP_B20g01644* | 0.10669522 | 0.89583569 | 0.11910133 |
| *CP_A09g00610* | *CP_B12g01171* | 0.02909064 | 0.09860332 | 0.29502696 |
| *CP_B12g01171* | *CP_B19g01374* | 0.54484025 | 1.99612681 | 0.27294872 |
| *CP_A10g00948* | *CP_B20g01408* | 0.02487201 | 0.06150986 | 0.40435806 |
| *CP_B11g00306* | *CP_B20g01408* | 0.24030845 | 0.66543902 | 0.36112769 |
| *CP_A10g00948* | *CP_B11g00306* | 0.24090808 | 0.68358076 | 0.3524208 |
| *CP_A09g00312* | *CP_B19g01626* | 0.37538719 | 1.31636229 | 0.28517012 |
| *CP_B12g01534* | *CP_B19g01626* | 0.39318816 | 1.4345107 | 0.27409217 |
| *CP_A08g00491* | *CP_B13g01720* | 0.00882623 | 0.08350224 | 0.10570049 |
| *CP_A06g00542* | *CP_B18g01335* | 0.00701431 | 0.09968567 | 0.07036431 |
| *CP_A02g00035* | *CP_B17g02638* | 0.13634648 | 0.6070406 | 0.2246085 |
| *CP_A05g01681* | *CP_B17g02638* | 0.00941632 | 0.05569568 | 0.16906731 |
| *CP_B13g00039* | *CP_B17g02638* | 0.12063863 | 0.66267157 | 0.1820489 |
| *CP_A02g00035* | *CP_A05g01681* | 0.12738884 | 0.58619488 | 0.21731483 |
| *CP_A02g00035* | *CP_B13g00039* | 0.01650479 | 0.10780857 | 0.15309351 |
| *CP_A03g01905* | *CP_B15g00859* | 0.02254268 | 0.09959361 | 0.22634667 |
| *CP_A03g01905* | *CP_B15g00859* | 0.02254268 | 0.09959361 | 0.22634667 |
| *CP_A02g00417* | *CP_A05g01179* | 0.12407212 | 0.59108665 | 0.20990512 |
| *CP_A02g00417* | *CP_B13g00551* | 0.02580395 | 0.10200882 | 0.25295806 |
| *CP_A02g00417* | *CP_B17g02220* | 0.1054899 | 0.59226829 | 0.17811167 |
| *CP_A09g00312* | *CP_B12g01534* | 0.01094767 | 0.0548645 | 0.19954017 |
| *CP_A07g00385* | *CP_A10g00582* | 0.19976423 | 0.58183291 | 0.34333608 |
| *CP_A07g00385* | *CP_B14g01310* | 0.01046094 | 0.08777127 | 0.11918412 |
| *CP_A07g00385* | *CP_B22g00881* | 0.13478282 | 0.54266677 | 0.24837124 |
| *CP_B14g01310* | *CP_B22g00881* | 0.1309824 | 0.53322972 | 0.24563972 |
| *CP_A05g01945* | *CP_A08g01689* | 0.14100508 | 0.74704762 | 0.18874978 |
| *CP_A05g01945* | *CP_B17g00516* | 0.04142626 | 0.1666133 | 0.24863718 |
| *CP_A10g00582* | *CP_B22g00881* | 0.09212046 | 0.16380335 | 0.56238448 |
| *CP_B04g01969* | *CP_A16g00063* | 0.02430105 | 0.17393313 | 0.13971489 |
| *CP_A10g01363* | *CP_B20g01155* | 0.03791678 | 0.11149786 | 0.34006731 |
| *CP_A01g01358* | *CP_B12g00251* | 0.05527681 | 0.18904552 | 0.29239945 |
| *CP_A08g01689* | *CP_B17g00516* | 0.1491898 | 0.73951429 | 0.20174025 |
| *CP_A06g02002* | *CP_B19g01374* | 0.01205329 | 0.07428134 | 0.16226541 |
| *CP_A05g01179* | *CP_B17g02220* | 0.00407402 | 0.08002253 | 0.05091093 |
| *CP_B13g00551* | *CP_B17g02220* | 0.09213513 | 0.61688448 | 0.14935556 |
| *CP_A05g01179* | *CP_B13g00551* | 0.08942241 | 0.61902564 | 0.14445671 |
| *CP_A03g02030* | *CP_B15g00697* | 0.0143851 | 0.12809956 | 0.11229623 |

## Supplementary Table 4. miRNA prediction for HD-ZIP genes (see separate file).

## Supplementary Table 5. *Cis*-acting element identified in the promotor region (see separate file).

## Supplementary Table 6. *Cis*-acting element types and numbers (see separate file).

## Supplementary Table 7. Statistical analysis using multiple comparisons for qPCR expression levels between two types of treatment.

| **Gene** | **Dunnett's multiple comparisons test** | **Mean Diff.** | **95.00% CI of diff.** | **Below threshold?** | **Summary** | **Adjusted P value** |
| --- | --- | --- | --- | --- | --- | --- |
| *DACA009322* | Control-R vs. NR | -0.77 | -0.9225 to -0.6175 | Yes | **** | <0.0001 |
|  | Control-R vs. CR | 0.05 | -0.1025 to 0.2025 | No | ns | 0.8312 |
|  | Control-R vs. Control-L | 0 | -0.1525 to 0.1525 | No | ns | >0.9999 |
|  | Control-R vs. NL | -0.425 | -0.5775 to -0.2725 | Yes | **** | <0.0001 |
|  | Control-R vs. CL | -0.2075 | -0.3600 to -0.05501 | Yes | ** | 0.0061 |
| *DACA012489* | Control-R vs. NR | 0.8175 | 0.6114 to 1.024 | Yes | **** | <0.0001 |
|  | Control-R vs. CR | 0.155 | -0.05106 to 0.3611 | No | ns | 0.1818 |
|  | Control-R vs. Control-L | 0 | -0.2061 to 0.2061 | No | ns | >0.9999 |
|  | Control-R vs. NL | 0.38 | 0.1739 to 0.5861 | Yes | *** | 0.0004 |
|  | Control-R vs. CL | 0.8925 | 0.6864 to 1.099 | Yes | **** | <0.0001 |
| *DACA002429* | Control-R vs. NR | 0.01 | -0.1256 to 0.1456 | No | ns | 0.9997 |
|  | Control-R vs. CR | 0.6325 | 0.4969 to 0.7681 | Yes | **** | <0.0001 |
|  | Control-R vs. Control-L | 0 | -0.1356 to 0.1356 | No | ns | >0.9999 |
|  | Control-R vs. NL | -0.495 | -0.6306 to -0.3594 | Yes | **** | <0.0001 |
|  | Control-R vs. CL | 0.7575 | 0.6219 to 0.8931 | Yes | **** | <0.0001 |
| *DACA020351* | Control-R vs. NR | -1.805 | -2.042 to -1.568 | Yes | **** | <0.0001 |
|  | Control-R vs. CR | -1.363 | -1.600 to -1.125 | Yes | **** | <0.0001 |
|  | Control-R vs. Control-L | 0 | -0.2374 to 0.2374 | No | ns | >0.9999 |
|  | Control-R vs. NL | 0.0575 | -0.1799 to 0.2949 | No | ns | 0.94 |
|  | Control-R vs. NR | -0.38 | -0.7522 to -0.007751 | Yes | * | 0.0445 |
| *CP_B22g00881* | Control-R vs. CR | -0.9675 | -1.340 to -0.5953 | Yes | **** | <0.0001 |
|  | Control-R vs. Control-L | 0 | -0.3722 to 0.3722 | No | ns | >0.9999 |
|  | Control-R vs. NL | -0.4925 | -0.8647 to -0.1203 | Yes | ** | 0.0077 |
|  | Control-R vs. CL | 0.12 | -0.2522 to 0.4922 | No | ns | 0.8398 |
|  | Control-R vs. NR | -1.018 | -1.234 to -0.8006 | Yes | **** | <0.0001 |
| *CP_B11g00306* | Control-R vs. CR | -1.355 | -1.572 to -1.138 | Yes | **** | <0.0001 |
|  | Control-R vs. Control-L | 0 | -0.2169 to 0.2169 | No | ns | >0.9999 |
|  | Control-R vs. NL | 0.2525 | 0.03558 to 0.4694 | Yes | * | 0.0196 |
|  | Control-R vs. CL | -1.143 | -1.359 to -0.9256 | Yes | **** | <0.0001 |
|  | Control-R vs. NR | -0.7725 | -1.548 to 0.003449 | No | ns | 0.0512 |
| *CP-A06g00792* | Control-R vs. CR | -4.503 | -5.278 to -3.727 | Yes | **** | <0.0001 |
|  | Control-R vs. Control-L | 0 | -0.7759 to 0.7759 | No | ns | >0.9999 |
|  | Control-R vs. NL | 0.68 | -0.09595 to 1.456 | No | ns | 0.0974 |
|  | Control-R vs. CL | 0.69 | -0.08595 to 1.466 | No | ns | 0.091 |
|  | Control-R vs. NR | 0.005 | -0.1694 to 0.1794 | No | ns | 0.9999 |
| *CP_A10g00662* | Control-R vs. CR | -0.235 | -0.4094 to -0.06061 | Yes | ** | 0.0066 |
|  | Control-R vs. Control-L | 0 | -0.1744 to 0.1744 | No | ns | >0.9999 |
|  | Control-R vs. NL | -0.195 | -0.3694 to -0.02061 | Yes | * | 0.0256 |
|  | Control-R vs. CL | -0.2225 | -0.3969 to -0.04811 | Yes | * | 0.0102 |
